# Revascularization of Left Anterior Descending Artery with Minimally Invasive Direct Coronary Artery Bypass Graft vs. Drug Eluting Stents: A Retrospective, Two-Center Study

**DOI:** 10.3390/jcm15051863

**Published:** 2026-02-28

**Authors:** Amit Gordon, Yaron Moshkovitz, Dmitry Pevni, Orr Sela, Nadav Teich, Mohammad Kakoush, Tomer Ziv-Baran, Yanai Ben-Gal

**Affiliations:** 1 Department of Cardiac Surgery, Tel Aviv Sourasky Medical Center, School of Medicine, Gray Faculty of Medical & Health Sciences, Tel-Aviv University, Tel-Aviv 6927846, Israel; amitgor@tlvmc.gov.il (A.G.); dimitrip@tlvmc.gov.il (D.P.); orrse@tlvmc.gov.il (O.S.); nadavt@tlvmc.gov.il (N.T.); mohammadk@tlvmc.gov.il (M.K.); 2 Department of Cardiothoracic Surgery, Assuta Ramat HaHayal Hospital, Joyce & Irving Goldman School of Medicine, Ben-Gurion University of the Negev, Beer Sheva 84105, Israel; mosko@assuta.co.il; 3 School of Public Health, Gray Faculty of Medical & Health Sciences, Tel-Aviv University, Tel-Aviv 6927846, Israel

**Keywords:** MIDCAB, CABG, DES, Cypher, PCI, IHD, LAD

## Abstract

**Background/Objectives:** Revascularization of the left anterior descending (LAD) artery can be achieved by either percutaneous coronary intervention (PCI) or coronary artery bypass grafting (CABG). Minimally invasive direct CABG (MIDCAB) enables LAD revascularization via a small thoracotomy without sternotomy or cardiopulmonary bypass. To compare long-term survival following LAD revascularization by MIDCAB or following PCI using drug-eluting stents (DES), of the historic cohort we reported in 2006. **Methods:** Data were assessed of 272 patients who underwent LAD PCI with DES, and 104 patients who underwent MIDCAB using the left internal thoracic artery (LITA) to LAD, in two major centers, between May 2002 and December 2003. Matching for age, sex, and extent of coronary disease yielded two balanced groups of 83 patients each. **Results:** Baseline characteristics were similar with a mean age ± standardized difference (SD) of 64.70 ± 12.52 of the MIDCAB group vs. 63.59 ± 12.06 of the Cypher group and an identical male to female ratio of 66 to 83 (79.5%), except for a higher prevalence of EF < 35% in the MIDCAB group and prior PCI in the DES group. Thirty-day mortality was 1.1% after MIDCAB and 0% after DES-PCI (*p* = 0.11). At 2 years, the proportion of recurrent angina was lower after MIDCAB (8.4% vs. 35%; *p* < 0.001), as was the proportion of re-interventions (3.6% vs. 16.8%; *p* = 0.005). Over a mean follow-up of 16 years, 10-year survival was 77.1 ± 4.6% for the MIDCAB and 81.0 ± 4.3% for the DES group (*p* = 0.48). The rates of 20-year survival were 60.2 ± 5.4% and 56.1 ± 5.5%, respectively (*p* = 0.73). In multivariable analysis, advanced age and prior myocardial infarction independently predicted mortality while treatment with MIDCAB showed a trend toward improved survival (*p* = 0.053). **Conclusions:** Long-term survival rates after LAD revascularization with MIDCAB and after DES-PCI were comparable. MIDCAB demonstrated a non-significant trend toward lower mortality. Limitations include the retrospective design and lack of detailed late event adjudication.

## 1. Introduction

Given the pivotal role of the left anterior descending (LAD) artery in myocardial perfusion, percutaneous coronary intervention (PCI) for this vessel typically involves the use of drug-eluting stents (DESs). Compared with bare-metal stents, DESs provide superior patency and significantly lower rates of restenosis and repeat revascularization [1]. However, certain LAD lesions remain suboptimal for PCI. These include long, tortuous, or heavily calcified segments; ostial or bifurcation lesions; sites where stenting may jeopardize adjacent branches; regions of in-stent restenosis; and chronic total occlusions [2]. Although more invasive than PCI, surgical revascularization continues to represent the standard of care for patients with multivessel or complex coronary artery disease [3], largely due to the proven long-term durability of left internal thoracic artery (LITA) grafting to the LAD [4].

Minimally invasive direct coronary artery bypass (MIDCAB) is performed through a small anterolateral thoracotomy, thus providing direct access to the LAD, which is revascularized using the LITA as an in situ graft [5]. The operation is typically carried out off-pump, without the use of extracorporeal circulation, and is rarely extended beyond the LAD territory [6]. At our institution, MIDCAB is primarily offered to patients requiring isolated LAD revascularization or with complex LAD lesions unsuitable for PCI; or patients deemed high-risk for conventional surgery due to significant comorbidities, frailty, advanced age, osteoporosis, or impaired mobility.

Following the introduction of DES in the early 2000s, we sought to compare early and mid-term outcomes in patients with significant LAD disease treated either by DES implantation or by MIDCAB. In one of the first direct comparisons between surgical revascularization and DES-based PCI, 376 patients were evaluated between May 2002 and December 2003. The final analysis included 83 matched pairs who were followed for a median of 22 months after undergoing either MIDCAB or LAD stenting with a Cypher DES. As reported by our group in 2006 [7], early and late mortality rates were similar between the two strategies, whereas the DES group demonstrated higher rates of reintervention and recurrent angina. In the current study, we re-examined this original cohort to evaluate long-term mortality and identify predictors of late outcomes, with a substantially extended mean follow-up approaching 20 years.

## 2. Materials and Methods

### 2.1. Patients and Methods

The study population comprised 376 patients with coronary artery disease treated at two major medical centers between May 2002 and December 2003. Patients underwent either PCI with a Cypher DES or MIDCAB using an in situ LITA graft to the LAD. The data were accessed for research purposes on 1–3 May 2025. The authors had access to identifying data during data collection.

Initially, 272 patients were included in the PCI group and 104 in the surgical group. The groups were subsequently matched for age, sex, and number of diseased vessels. We previously reported early and mid-term outcomes of the matched groups [7]. In the present analysis, both groups were re-examined for long-term survival, with late survival data obtained from the Israeli National Registry. The study protocol was approved by the institutional review board. Informed consent was waived due to the retrospective nature of the study.

During the study period, selection criteria for surgery versus PCI were mainly technical. In principle, there was a preference to refer patients for surgery for the following reasons:

- Comorbid diseases such as diabetes mellitus and renal failure;
- In-stent restenosis or thrombosis of a coronary artery;
- Complex type C lesions (calcified coronary arteries, lesion length exceeding 20 mm, twisted arteries, suspicion of a thrombus in an artery), or bifurcation lesion involving a major diagonal branch;
- Cypher drug-eluting stents were not available;
- Complete occlusion;
- Patient’s or cardiologist’s preference.

PCI procedures were performed using sirolimus-eluting stents (Cypher, Cordis, Johnson & Johnson, Miami Lakes, FL, USA) following standard balloon pre-dilation. All patients received aspirin 325 mg before and after the procedure and a 300 mg loading dose of clopidogrel, followed by 75 mg daily for at least three months. Intravenous heparin was administered intra-procedurally. In most patients, a single Cypher stent was implanted in the LAD, although multiple stents were deployed for longer lesions. In two patients with bifurcation disease, both the LAD and diagonal branch were treated.

MIDCAB procedures were performed through a small left anterolateral thoracotomy, typically in the fourth intercostal space. The LITA was harvested under direct vision and used as an in-situ graft to the LAD. Heparin was administered at a dose of 200 U/kg to achieve an activated clotting time of about 400 s. The anastomosis was performed on a beating heart using a running 7-0 polypropylene suture (Norgrine Limited, Uxbridge, UK), without using cardiopulmonary bypass, yet utilizing local mechanical stabilization and CO_2_ insufflation for optimal visualization. Heparin was reversed with protamine, and the wound was closed in a standard manner. Postoperatively, all the patients received an intravenous infusion of isosorbide dinitrate (Isoket, 4–20 mg/h) for the first 24–48 h.

### 2.2. Statistical Analysis

In the current study, similar statistical analyses to the previous one were applied [7]. Data were expressed as number and proportions. The two groups were matched in the previous study using age, sex, and extent of coronary artery disease.

Categorical variables were compared between groups using the Chi-square or Fisher’s exact test, as appropriate survival was assessed using Kaplan–Meier curves, and differences between groups were evaluated with the log-rank test. Univariate Cox regression was performed to assess crude associations between each variable and all-cause mortality. Multivariable Cox regression was applied to evaluate the independent association between revascularization strategy and mortality, adjusting for potential confounders. In order to comply with the rule of thumb of ten events-per-variable, the following variable selection strategy was applied. The multivariable model was constructed in two stages. In the first block, revascularization technique, age, and sex were entered. In the second block, additional covariates were considered using a forward selection method, with inclusion based on a Wald test *p*-value < 0.1. Candidate variables included congestive heart failure, diabetes mellitus, hypertension, peripheral vascular disease, hyperlipidemia, chronic obstructive pulmonary disease, chronic renal failure, prior myocardial infarction (MI), acute MI, ejection fraction < 35%, left main disease, prior PCI, intra-aortic balloon pump use, emergency presentation, and redo surgery. The partial residuals were calculated and plotted against the survival time to test the proportional hazards assumption.

All tests were two-tailed, and statistical significance was defined as *p* < 0.05. Analyses were conducted using SPSS (Version 31.0.1.0) Statistics for Windows (IBM Corp., Armonk, NY, USA).

## 3. Results

### 3.1. Early and Midterm Results

As previously reported [7], after matching 376 patients for age, sex, and the extent of coronary artery disease, the two groups with 83 patients each were largely comparable. However, left ventricular EF < 35% was more common in the MIDCAB group, while a history of prior PCI was more frequent in the DES group (Table 1). There were no significant differences between the groups in the number of diseased vessels and in the presence of left main lesions (Table 1). Totally occluded LADs were more prevalent in the MIDCAB group.

As previously reported [7], 30-day mortality was 1.1% in the MIDCAB group and 0% in the DES group. At two-year follow-up (Table 2), which included a structured telephone survey, Kaplan–Meier survival was 93% after MIDCAB and 98% after DES (*p* = 0.11). Recurrent angina occurred in 8.4% of MIDCAB patients versus 35% of DES patients (*p* < 0.001). There were 3 reinterventions in the MIDCAB cohort and 14 reinterventions on the DES patients. Repeat LAD revascularization was necessary in one MIDCAB patient and seven DES patients (*p* = 0.029).

At two years, the incidence of major adverse cardiac events (MACE: cardiac death, MI, or reintervention) was significantly higher after DES implantation than after MIDCAB (20.5% vs. 7.2%; *p* = 0.013). Angina-free survival at two years was 87.4% for MIDCAB versus 57% for DES (*p* = 0.002). Independent predictors of MACE at two years included DES treatment (hazard ratio (HR) 4.1; 95% confidence interval (CI) 1.26–13.16), multivessel disease (HR 4.3; 95% CI 1.44–13.16), and prior PCI (HR 4.36; 95% CI 1.28–14.90). On multivariable Cox regression controlling for baseline differences, DES revascularization remained the sole independent predictor of both recurrent angina (HR 6.17; 95% CI 2.46–15.4) and reintervention (HR 8.26; 95% CI 1.68–40).

### 3.2. Late Results

**Figure 1:**
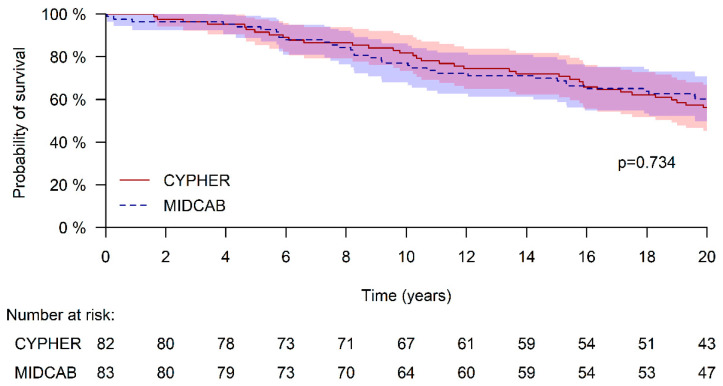
Long-term survival. Cypher—Treated with Cypher DES. MIDCAB—Minimally Invasive Direct Coronary Artery Bypass.

The mean follow-up duration in the current analysis was 16 years. Of note, one patient in the Cypher group could not be tracked in the Israeli national registry. Survival data were available for 99% of the study population, with only one DES-treated patient missing from the National Registry. At 10 years, survival was similar between the MIDCAB and DES groups (77.1% ± 4.6% vs. 81.0% ± 4.3%, *p* = 0.48). This similarity persisted at 20 years, with survival rates of 60.2% ± 5.4% and 56.1% ± 5.5%, respectively (*p* = 0.73; Figure 1).

In univariate analysis, factors significantly associated with increased overall mortality included older age (HR 1.096; 95% CI 1.071–1.121), congestive heart failure (HR 2.63; 95% CI 1.14–6.11), chronic renal failure (HR 3.49; 95% CI 1.59–7.68), three-vessel disease at the index procedure (HR 2.91; 95% CI 1.58–5.53), and prior cardiac surgery (redo operation) (HR 12.36; 95% CI 2.86–53.40).

Multivariable Cox regression showed a trend (still non-significant) towards lower mortality for patients treated by MIDCAB surgery (Table 3).

## 4. Discussion

MIDCAB was first performed by Vasilii Kolesov in 1967 [8]. Although the technique was largely abandoned for several decades, it regained interest in the mid-1990s as a safe and reproducible surgical option [9,10,11]. In contemporary practice, MIDCAB may be performed using thoracoscopic or robotic-assisted approaches, both of which have gained broad acceptance as reliable and effective minimally invasive revascularization strategies [12,13].

This study builds upon our 2006 publication, which was among the first to compare early outcomes of myocardial revascularization using DES (Cypher) to LAD versus surgical revascularization with an internal thoracic (LITA) graft to the LAD via MIDCAB [7]. That report focused on two historic matched cohorts of 83 patients each, balanced for age, sex, and the extent of coronary artery disease. The findings suggested a clinical advantage for MIDCAB in terms of angina relief and lower rates of repeat revascularization, primarily in the LAD territory. The incidence of MACE was significantly higher in the DES group, while treatment with DES was an independent predictor for re-angina and re-interventions. During the median follow-up of almost 2 years, no difference was observed in overall survival between the groups.

In the present analysis, we revisited the original cohort of 83 matched patient pairs to assess the long-term survival outcomes, extending up to 20 years. Notably, both groups demonstrated excellent long-term results. Ten-year survival was 81.0% ± 4.3% in the DES group and 77.1% ± 4.6% in the MIDCAB group (*p* = 0.48). At 20 years, survival was respectively 56.1% ± 5.5% and 60.2% ± 5.4% (*p* = 0.734). Multivariable analysis revealed that age and old MI at the index procedure were independently associated with increased mortality. In addition, treatment by MIDCAB demonstrated a trend (non-significant) towards lower mortality (HR 0.615 95% CI 0.376–1.007; *p* = 0.053).

Both MIDCAB and PCI are well-established revascularization strategies. While anatomical suitability is critical, treatment selection is equally influenced by a patient’s overall health, comorbidities, and clinical presentation. Differences between the two approaches are most evident across clinical settings, stable coronary artery disease, unstable angina, and ST-elevation MI (STEMI), which vary widely in procedural risk. PCI mortality ranges from <1% in stable patients to 30–50% in high-risk acute presentations, whereas MIDCAB mortality is typically lower, about 0.2% in stable patients and up to 5% in selected high-risk patients [14,15,16,17].

Long-term survival is largely determined by baseline characteristics and the presentation at the time of revascularization. After PCI, 10-year survival averages 80–90% in elective patients but falls below 50% in patients with STEMI and additional risk factors such as left ventricular dysfunction or diabetes mellitus [3,18,19,20,21]. Although long-term data for MIDCAB are more limited, available evidence suggests survival comparable to that of an age- and sex-matched general population, with 10-year survival rates ranging from 80% to 90% [22,23].

Comparative evidence of the safety and efficacy of LAD revascularization by PCI versus MIDCAB remains limited. In a study by Patel et al., 158 propensity-matched pairs with proximal LAD lesions underwent either second-generation DES implantation or MIDCAB. After nine years of follow-up, overall survival was similar between the groups; however, LAD reintervention was more frequent in the PCI cohort (13.4% vs. 2.5%, *p* < 0.0001) [24]. Similarly, a meta-analysis by Wang et al. including 941 patients, found no significant differences in early or one-year survival between patients treated with PCI (BMS or DES) or MIDCAB. Nonetheless, recurrent angina was less common after MIDCAB (12.9% vs. 28.7%; odds ratio (OR) 2.86; 95% CI 1.70–4.81; *p* < 0.0001). Also less common was the incidence of MACE, at both 6 months (9.3% vs. 18.2%; OR 2.12; 95% CI 1.19–3.79; *p* = 0.0009) and 1 year (15.4% vs. 23.4%; OR 1.84; 95% CI 1.21–2.78; *p* = 0.004) [25].

Blazek et al. compared outcomes in 110 propensity-matched pairs, between those who underwent PCI to the LAD with bare-metal stents and those who underwent MIDCAB using a LITA–LAD graft. At 10-year follow-up, there was no significant difference in the composite endpoint of death, MI, or target vessel revascularization (47% vs. 36%; *p* = 0.12). However, target vessel revascularization alone was significantly more frequent after PCI (34% vs. 11%; *p* < 0.01) [26,27]. In another study, 77 propensity-matched pairs were selected from a historical cohort of 2206 patients who underwent PCI and were treated with either DES implantation or MIDCAB. At 3-year follow-up, there were no significant differences in major adverse cardiac or cerebrovascular events, including target vessel revascularization (2.6% vs. 5.2%; *p* = 0.43) [28]. Similarly, Stanislawski et al. analyzed 111 propensity-matched pairs treated with either LAD DES-PCI or MIDCAB. While early and 5-year survival were comparable, the MIDCAB group experienced fewer MIs and target vessel reinterventions [29].

Our current analysis aligns with prior studies showing comparable survival after MIDCAB or PCI, alongside consistently lower rates of MACE and recurrent angina following MIDCAB. However, despite the extended follow-up, the present report is limited to crude all-cause survival, as data on cardiac-specific mortality and MACE were available only from the original 2-year analysis. Such information might have revealed additional distinctions between the two strategies. Also, due to the prolonged time interval since the initial analysis was performed, it was not possible to retrieve the specific matching between each patient and their corresponding counterpart in the other group; therefore, the analysis was conducted on the two patient groups as a single combined cohort. Despite matching, the groups remained imbalanced with respect to prior PCI and ejection fraction, with small but statistically significant differences. These residual differences may have influenced late outcomes. Importantly, treatment allocation was not random with high probability for selection bias, and substantial determinants differentiating the groups may still be hidden. As there were only 68 patients who died through the follow-up period and in order to comply with the rule of thumb of ten events-per variable, only seven variables were able to be entered into the regression. Data were also absent on lesion complexity, completeness of revascularization, and staged PCI for non-LAD disease. Treatment was determined by different surgical and interventional teams, without a unified institutional protocol, thus introducing potential selection bias. Moreover, PCI in this cohort utilized only first-generation DES, warranting cautious interpretation as an historic comparison, and cannot be generalized for current generation DES and long-term DAPT therapy. All MIDCAB procedures were performed by a single highly experienced surgeon, whereas multiple interventional cardiologists performed PCI, which may have influenced the outcomes. Despite matching, residual baseline differences persisted, particularly lower EF and higher rates of prior PCI in the MIDCAB group, which could have affected the long-term results.

## 5. Conclusions

In conclusion, this study supports the long-term safety and efficacy of both MIDCAB and DES-based PCI for LAD revascularization. Twenty-year survival was comparable following the investigated strategies. However, in multivariable analysis, treatment by the MIDCAB cohort showed a near-significant trend toward improved long-term survival.

## Figures and Tables

**Table 1:** Preoperative characteristics.

	ALL	DES	MIDCAB	SMD	*p* Value
	(*n* = 166)	(*n* = 83)	(*n* = 83)		
Male	132 (79.5%)	66 (79.5%)	66 (79.5%)	0	NA
Age (>70 years)	64 (38.6%)	32 (38.6%)	32 (38.6%)	0	NA
Diabetes mellitus	49 (29.7%)	25 (30.1%)	24 (28.9%)	0.026	0.860
Hypertension	85 (51%)	39 (47%)	46 (55.4%)	0.169	0.270
Peripheral vascular disease	10 (6.1%)	6 (7.2%)	4 (4.8%)	0.101	0.510
Hyperlipidemia	79 (47.9%)	43 (51.8%)	36 (43.4%)	0.169	0.270
COPD	7 (4.2%)	3 (3.6%)	4 (4.8%)	0.06	0.700
CHF	9 (5.5%)	3 (3.6%)	6 (7.2%)	0.16	0.300
CRF	8 (4.8%)	3 (3.6%)	5 (6.0%)	0.113	0.470
Old myocardial infarction	52 (31.5%)	24 (28.9%)	28 (33.7%)	0.104	0.500
Acute myocardial infarction	11 (6.6%)	3 (3.6%)	8 (9.6%)	0.244	0.120
Ejection fraction < 35%	7 (4.3%)	1 (1.2%)	6 (7.2%)	0.303	0.053
Left main disease	7 (4.2%)	2 (2.4%)	5 (6.0%)	0.181	0.274
1-vessel disease	86 (51.8%)	43 (51.8%)	43 (51.8%)	0	>0.999
2- or 3-vessel disease	80 (48.2%)	40 (48.2%)	40 (48.2%)	0	
1-vessel disease, treated	164 (98.7%)	81 (97.6%)	83 (100%)	0.222	0.497
2-vessel disease, treated	2 (1.2%)	2 (2.4%)	0 (0%)	0.222	
Total occlusion	22 (13.2%)	2 (2.4%)	20 (24.1%)	0.675	<0.001
S/p PTCA	66 (39.7%)	30 (36.1%)	36 (43.4%)	0.148	0.340
Prior PCI to LAD + stent	32 (19.2%)	21 (25.3%)	11 (13.2%)	0.309	0.043
ISR-LAD	20 (12.1%)	13 (15.9%)	7 (8.4%)	0.223	0.144
IABP	0	0	0	0	NA
Emergency	3 (1.8%)	2 (2.4%)	1 (1.2%)	0.091	0.560
Redo	2 (1.2%)	0	2 (2.4%)	0.222	0.150

COPD, Chronic Obstructive Pulmonary Disease; CHF, Congestive Heart Failure; CRF, Chronic Renal Failure; PTCA, Percutaneous Transluminal Coronary Angioplasty; PCI, Percutaneous Coronary Intervention; LAD, Left Anterior Descending; ISR, In Stent Restenosis; IABP, Intra-Aortic Balloon pump; NA, Not Applicable; SMD, Standardized Mean Difference.

**Table 2:** Post procedural outcomes up to 22 months.

	ALL	CYPHER DES	MIDCAB	*p* Value
Perioperative CVA	3 (1.8%)	1 (1.2%)	2 (2.4%)	>0.999
Perioperative MI	1 (0.6%)	1 (1.2%)	0	0.497
RE explore, bleeding	0	0	0	NA
RE-Angina	36 (21.8%)	29 (35.4%)	7 (8.4%)	<0.001
Incomplete revascularization	11 (6.7%)	6 (7.3%)	5 (6.0%)	0.739
Postoperative PCI	21 (12.7%)	16 (19.5%)	5 (6.0%)	0.007
LAD complication	13 (7.9%)	12 (14.6%)	1 (1.2%)	0.001
Re-intervention numbers	13 (7.9%)	11 (13.4%)	2 (2.4%)	0.009
LAD-reintervention numbers	8 (4.8%)	7 (8.4%)	1 (1.2%)	0.029
LATE MI	5 (3.0%)	4 (4.9%)	1 (1.2%)	0.210

DES, Drug Eluting Stent; CVA, Cerebrovascular Accident; MI, Myocardial Infarction; PCI, Percutaneous Coronary Intervention; LAD, Left Anterior Descending. NA, Not Applicable.

**Table 3:** Multivariable analysis for late mortality.

Predictor	HR (95% CI)	*p* Value
MIDCAB Group	0.615 (0.376–1.007)	0.053
Age	1.110 (1.082–1.139)	<0.001
Female	0.686 (0.389–1.209)	0.192
COPD	2.818 (0.985–8.063)	0.053
CHF	2.099 (0.856–5.148)	0.105
OLD MI	1.711 (1.022–2.865)	0.041

MIDCAB, Minimally Invasive Direct Coronary Artery Bypass Grafting; COPD, Chronic Obstructive Pulmonary Disease; CHF, Congestive Heart Failure; MI, Myocardial Infarction.

## Data Availability

Some restrictions will apply. Data from this study are ethically and legally restricted by the Institutional Review Board of Tel Aviv Sourasky Medical Center to prevent compromising patient confidentiality. Any requests for data release should be addressed to Dr. Shmuel Kivity, Chairman of the Tel Aviv Sourasky Medical Center Institutional Review Board (IRB)/Ethics (Helsinki) Committee at: allergy@tlvmc.gov.il.

## Abbreviations

The following abbreviations are used in this manuscript:

LAD	Left Anterior Descending Artery
DES	Drug Eluting Stents
BMS	Bare Metal Stents
MIDCAB	Minimally Invasive Direct Coronary Artery Bypass Surgery
PCI	Percutaneous Coronary Intervention
EF	Ejection Fraction
LITA	Left Internal Thoracic Artery
HR	Hazard Ratio
MACE	Major Adverse Cardiac Events
MI	Myocardial Infarction
